# Documenting participant Consent in archival records: Aligning yesterday's practices with today's processes

**DOI:** 10.12688/hrbopenres.14254.1

**Published:** 2025-10-15

**Authors:** Nimitha Kuriakose, Heidi Annuk, Saoirse Duggan, Oliver Carroll, Michael Kerin, Nicola Miller

**Affiliations:** 1 Discipline of Surgery, Lambe Institute for Translational Research, University of Galway, Galway, County Galway, Ireland; 2 University of Galway, Galway, County Galway, Ireland

**Keywords:** Informed consent, Research reproducibility, Quality control, Biobank network, Ethical governance, Quality data, Biospecimen access, Data annotation and management

## Abstract

The Cancer Biobank collects, processes, stores and distributes biological specimens and associated data from consenting participants, under a joint data controllership agreement between the University of Galway and HSE West and North West. Working towards improved transparency and in line with the requirements of ISO 20387 Biobanking accreditation, the Cancer Biobank has supported clinical trials and translational research for over 20 years.

The process of consent acquisition has evolved since the earliest samples (and data) were collected for research in 1998. With the introduction of GDPR (General Data Protection Regulation) in 2018, a consent declaration was sought from the Health Research Consent Declaration Committee (HRCDC) in 2019 to continue to store personal data on participants recruited to the Cancer Biobank when versions 1 and 2 of the consent documentation were in use (April 1998 – October 2008). This work outlines the process of clarifying which versions of consent forms were used to recruit participants from 1998–2025.

The task of verifying consent documentation for 8,925 participants recruited to the Cancer Biobank between 1998 and 2025 required detailed manual checking, recording, and scanning of all paper and electronic consent forms. Among these participants, 65% had signed Cancer Biobank consent forms, 14% had other types of study-specific consent forms and 21% had no proof of consent on file.

Consent auditing is essential in biobanking to safeguard participant data, to maintain regulatory compliance and ethical research standards and to sustain public confidence in the use of irreplaceable biological resources. Applying modern compliance standards to archival biobank records has involved navigating incomplete and incompatible consent records, evolving ethical norms, upgrading privacy protections, managing operational challenges and securing appropriate regulatory approval, namely a consent waiver. This complex environment requires adaptive governance strategies balancing legal, ethical and scientific priorities for responsible public interest biobanking.

## Introduction

The Cancer Biobank, University of Galway operates under the joint data controllership of University of Galway and the HSE (Health Service Executive) West and North West healthcare region. Over the years and in line with practice guidance, the processes of obtaining informed participant consent have evolved according to changes in study requirements and developments in specimen processing and storage as outlined in Figure 1.

The earliest participant consent forms on record date from 1998, when ethical approval was granted for a study focusing on the inheritance of breast cancer predisposition genes in the west of Ireland. The aim of that study was to recruit women and men with breast cancer, or a family history of the disease, and non-cancer controls to participate in research to identify breast cancer predisposition genes and variants. In the subsequent decade, blood samples and family history data were collected from approximately 1000 breast cancer cases and 1000 non-cancer controls. In collaboration with the Breast Cancer Association Consortium ( https://www.ccge.medschl.cam.ac.uk/breast-cancer-association-consortium-bcac), samples from over 420,000 women representing more than 100 collaborating partners worldwide (including the Cancer Biobank) were used to define the genes that are most clinically useful for inclusion on genetic screening panels for the prediction of breast cancer risk. This has improved risk prediction models to guide genetic counselling, which has positively impacted women and their families worldwide ^1^ demonstrating the impact of prospective biobanking on the global knowledge base ^2– 5^.

The introduction of GDPR (General Data Protection Regulation) ( https://gdpr-info.eu) and the Health Research Regulations (HRR) in 2018 ( https://hrcdc.ie/wp-content/uploads/2024/08/Health_Research_Information_Principles-for-Consent.pdf) strengthened protection around participant consent in Irish biobanking, to ensure greater transparency, participant control, and ethical use of samples and data, while also setting higher standards for consent documentation including Participant Information Leaflets (PIL) and Informed Consent Forms (ICF). 2018 also marked the introduction of ISO:20387 General requirements for biobanking ^6^ to address gaps in biobanking quality, ethics and competency, to support reproducible research and trustworthy biobank operations worldwide. The same year also marked the publication of revised International Society for Biological and Environmental Repositories (ISBER) best practices, which were aimed at sharing successful strategies on providing fit-for-purpose specimens for research and to develop harmonised principles in the science and management of repositories and biobanks.

The Health Research Consent Declaration Committee (HRCDC) in Ireland was established following the enactment of the HRRs to address situations whereby obtaining explicit consent from participants in health research, to use their personal data, was not possible. In exceptional cases, this provides a legal mechanism to allow processing of personal data for health research without explicit consent, where it is deemed by the HRCDC that the public interest in conducting the research significantly outweighs the need for explicit consent.

These factors in combination provided the rationale for conducting this study, which aimed to clarify participant consenting documentation on file in the Cancer Biobank dating from 1998–2025 and to verify their regulatory compliance.

## Methods

This study involved examining hard copy consent documentation and electronic records of participants that were recruited to the Cancer Biobank, beginning in 1998. Ethical approval (CREC) to amend and expand the Cancer Biobank from its inception as a breast cancer study-specific cohort, to its current form, has necessitated amending and updating CREC (Clinical Research Ethics Committee), PIL (Participant Information Leaflets) and ICF documentation eleven times, most recently in 2024 corresponding to version 11 ^7^. Figure 1a demonstrates how the various CREC approvals and their amendments correspond to evolving biobank activities and specimen handling requirements, Figure 1b focuses on recent overarching legislative changes.

**Figure 1a.**
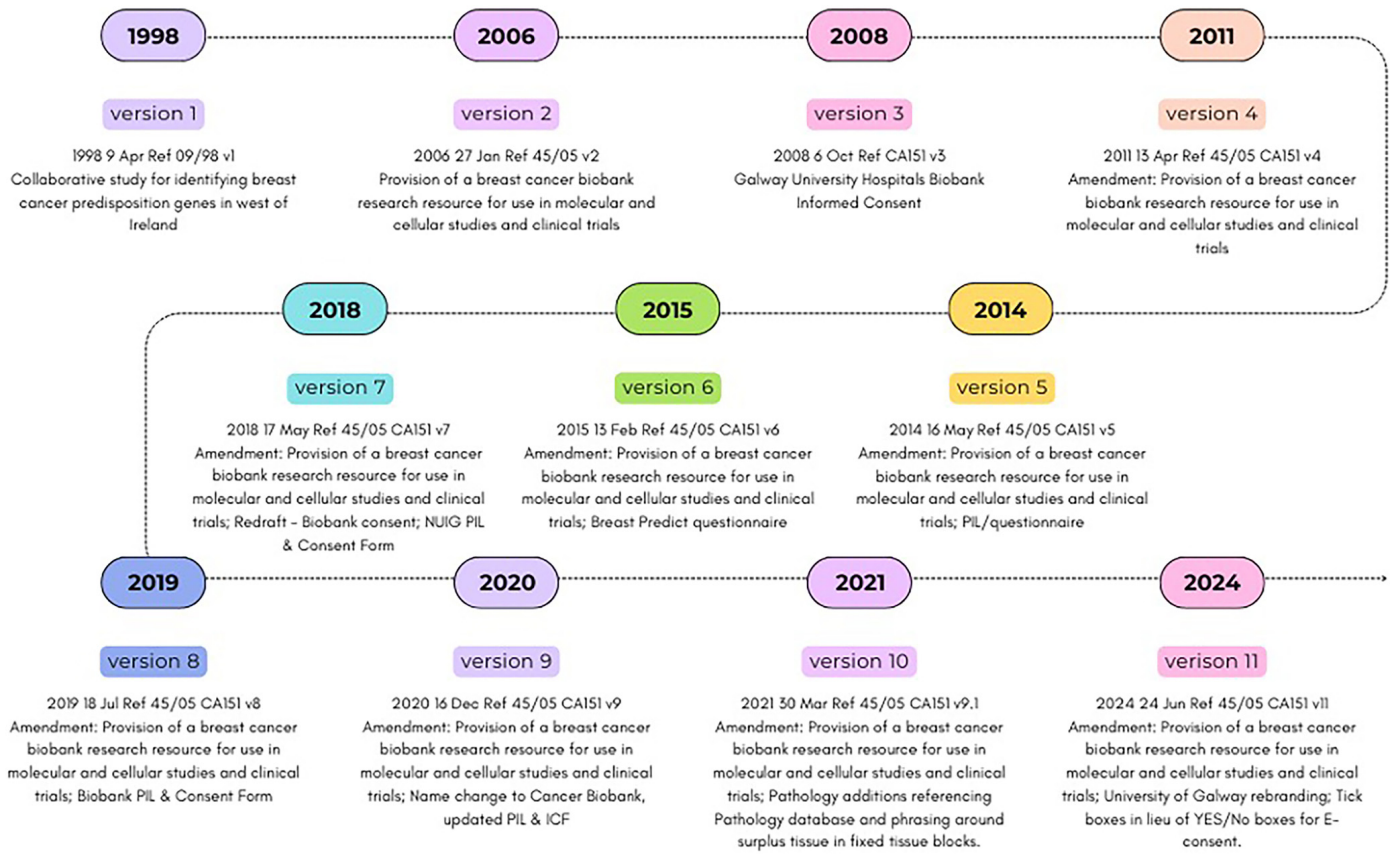
Cancer Biobank consent: CREC approval timelines and associated PIL and ICF versions.

**Figure 1b.**
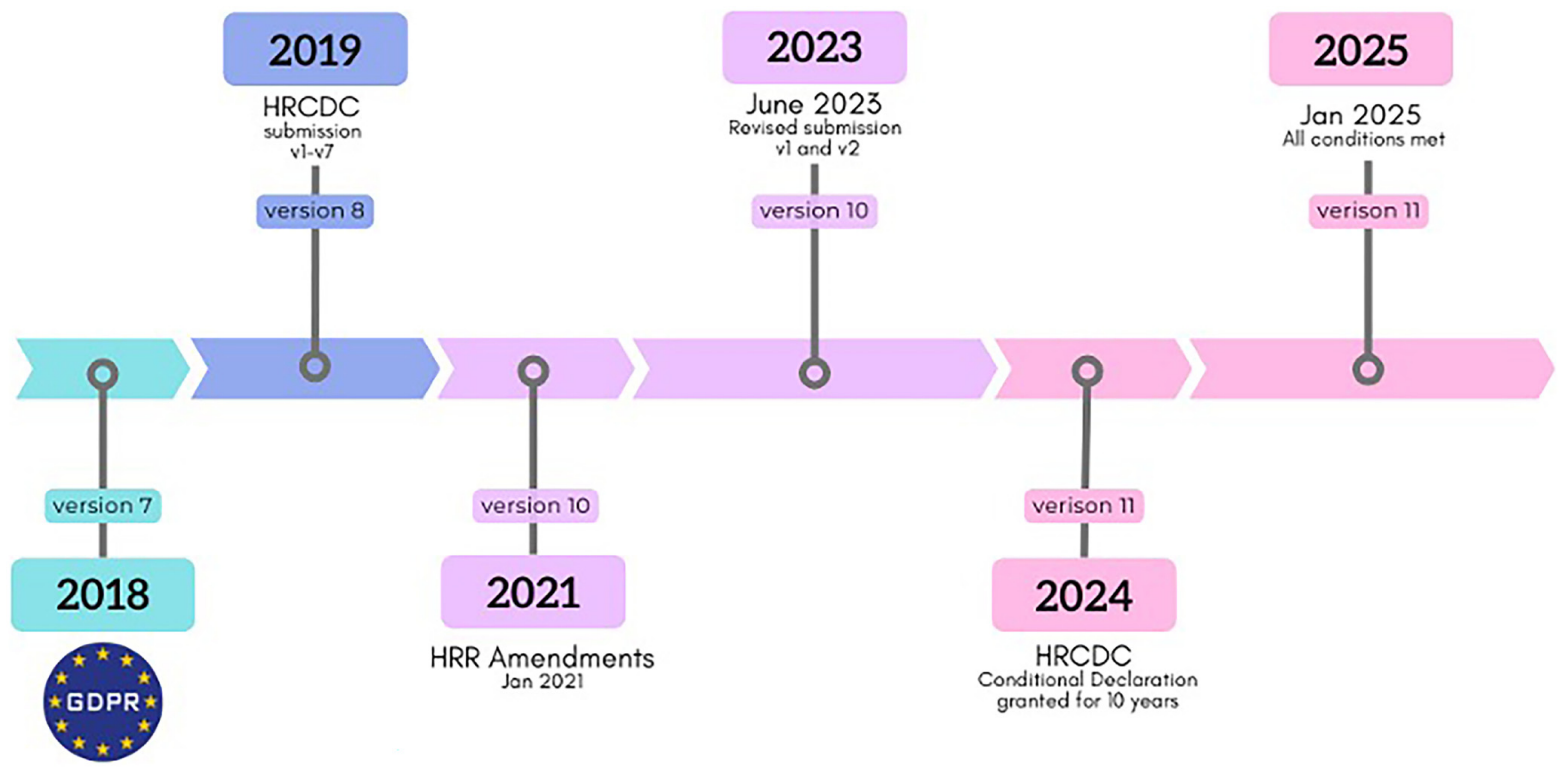
Cancer Biobank consent compliance, GDPR, HRR and consent declaration.

The process of obtaining participant consent has broadly followed a similar format, whereby following the consent conversation between prospective participants and a healthcare professional, specimens are returned to the Cancer Biobank laboratory, either accompanied by a signed copy of the ICF, or prior to 2019 when triplicate ICFs (and routine digitisation) were introduced, returned to the laboratory or to the patient’s chart. Hard copies of signed consent documentation are stored in locked filing cabinets in the Cancer Biobank.

In 2005, the Cancer Biobank implemented an MS SQL database software, to electronically manage specimen processing and storage data. Since then, the LIMS (Laboratory Information Management System) has been used to pseudonymise participant information. Specimens are labelled with LIMS-generated codes, with no identifying information appearing on laboratory documents including labels, worksheets etc. Any participants who had been recruited to the Cancer Biobank before the introduction of the LIMS were retrospectively added, and their specimens re-labelled as necessary. A consent declaration was sought from the Health Research Consent Declaration Committee (HRCDC) in 2019 to continue to store personal data on participants recruited to the Cancer Biobank when versions 1 and 2 of the consent documentation were in use (April 1998 – October 2008).

The process of accurately quantifying the numbers of patients recruited using sequential versions of the consent documentation, involved manual inspection of all participant records on file for the various versions of ICF and cross-checking each participant record with their electronic equivalent in the LIMS database. All verified copies of signed Cancer Biobank ICFs were digitised by scanning for upload to the LIMS system. To enhance data accessibility, the digitised consent forms were linked to the participant unique LIMS number, enabling proof of participant consent to be linked directly to the participant record. All hard copy files were then re-filed by ascending LIMS numbers and returned to the filing cabinets for storage. All participant records were then re-checked to verify the latest version of the ICF on file. This was relevant given the frequency that patients re-attended follow-up clinics and were reconsented when they donated follow-up samples. Any participants for whom a ‘later’ consent form was identified were excluded from counting in ‘earlier’ cohorts. All data was recorded in MS Excel version 16.0.

The HSE Evolve (electronic document management) system was used to identify which participants without record of consent were still living or deceased. Evolve was also searched for any record of biobank consent. The process of digitising medical charts to Evolve was begun in 2020. Patients that attended hospital or clinic from this year onward had both their current and archival medical charts digitised. If a patient had not been in attendance since 2020, their medical charts were archived in hard copy in the University Hospital Galway Library. If record of consent was not found on Evolve, verification of which consent form was used when these patients were being recruited, necessitated manual chart review. A subset of patient charts, whose consent was not found on Evolve were chosen for manual chart review. This involved accessing the HSE’s Patient Administration System (PAS) to identify the last date of attendance at a ward or clinic and cross-referencing with their medical chart identifier. Medical charts were either retrieved from local storage in the University Hospital Galway Library or requested from off-site storage in medical archives.

## Results

The task of verifying the presence/absence, version and quality (signed/tick-boxes) of consent documentation required painstaking checking, recording and scanning of all consent forms held on file either in hard-copy or electronically in the Cancer Biobank and is summarised in Figure 2a. This cohort amounted to 8925 participants (as of July 2025), was recruited between 1998–2025 and included 5852 (65%) participants with Cancer Biobank consent documentation (versions 1-11), 1218 (13%) participants with ‘other’ study consent documentation, and 1855 (21%) of participants with no proof of consent documentation either in hard copy or electronically. Further analysis revealed the distribution of versions of Cancer Biobank consent forms over time ( Figure 2b). To accurately represent the numbers of participants, rather than numbers of samples donated (tissue, blood, serial serum and/or plasma), the figures represent the most recent version of the signed Cancer Biobank consent form on file.

**Figure 2a.**
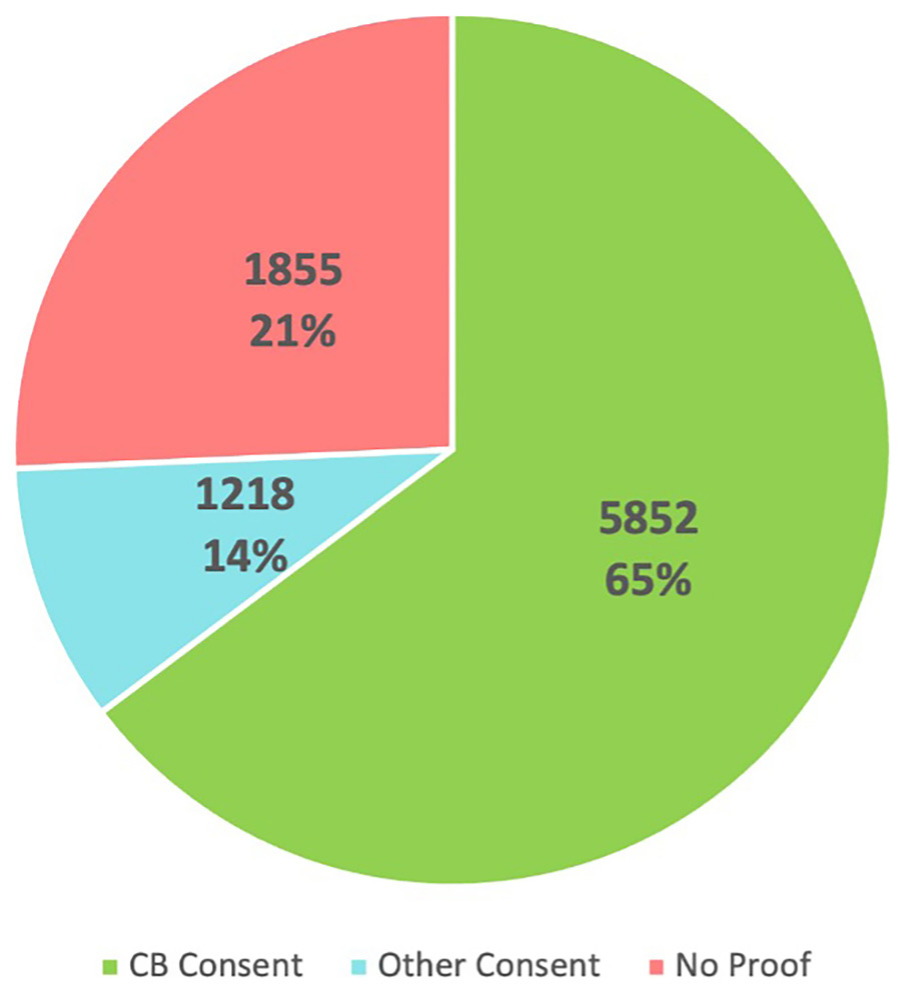
Participant consent documentation (n=8925) 1998–2025.

**Figure 2b.**
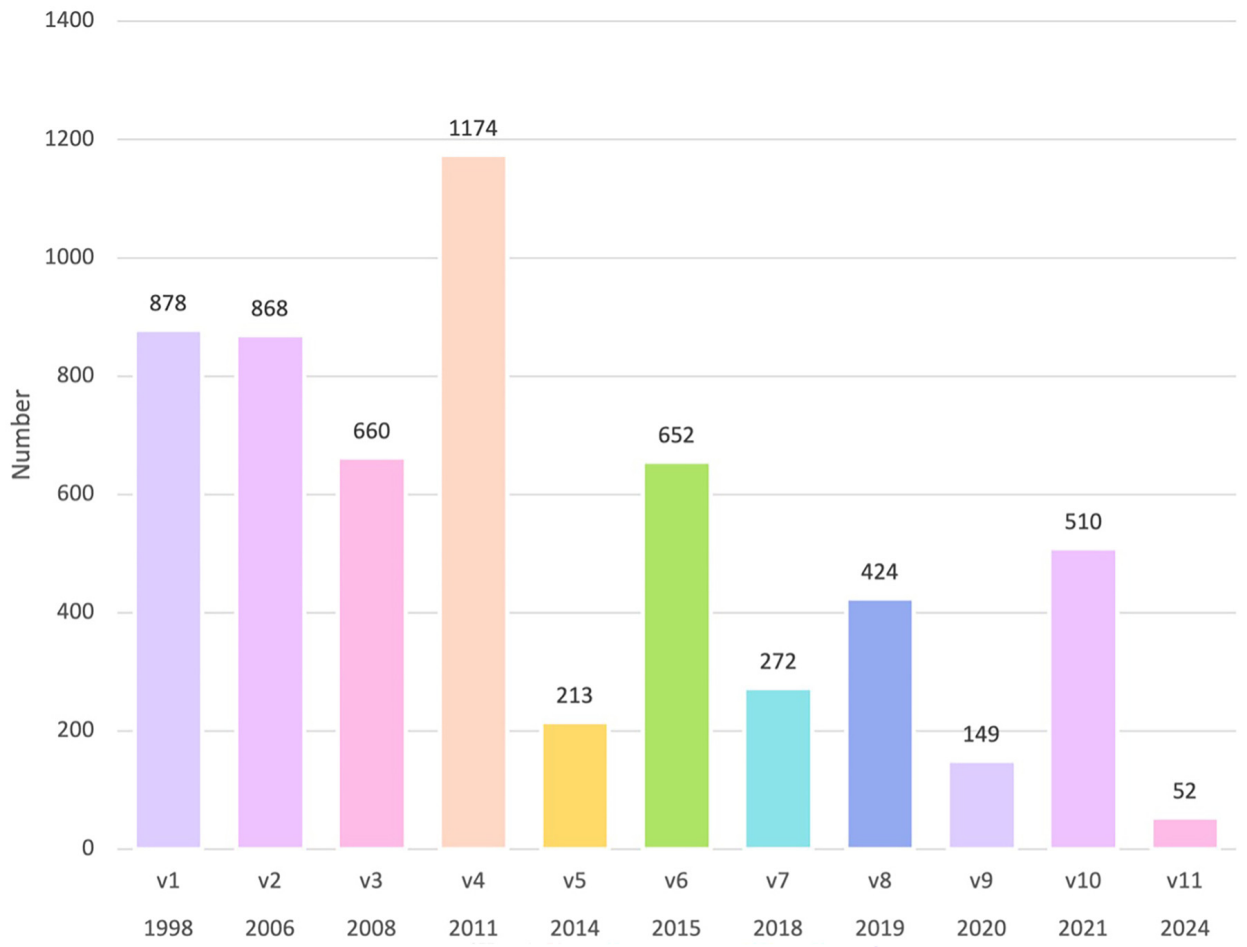
Versions (v1–v11) and numbers (n=5852) of Cancer Biobank ICFs on file from 1998–2025.

A chart review of participants who had been consented prior to the introduction of triplicate consent forms and routine scanning of consent documentation, identified that from January 2014–August 2019 (versions 5–8), 1561 participants had consented to biobanking (determined from the appropriate tick-box on the LIMS system). Of these 318 had no proof of consent. Follow-up manual searches in medical charts resulted in 15 (4.7%) signed forms being found.

In September 2020, an overview of participants recruited between 1998–2008 was conducted to establish if any participants for whom no proof of consent was identified, had subsequently died. This analysis revealed that 206 participants, without proof of consent, had died between recruitment and September 2020. These participants would not fall under GDPR. A total of 283 participants were recruited to the Cancer Biobank during the time when version 1 and version 2 consent documentation were in use, had no proof of consent and were still living.

Further internal auditing conducted by the Cancer Biobank governing body, the Clinical Research Development Office, to check the presence of valid consent on five randomly selected participants found that one participant had valid consent, one consent form had not been signed by the consenter, one had no proof of consent and two corresponded to erroneously created records, that have since been deleted.

A consent declaration to continue to store personal data on participants recruited to the Cancer Biobank when versions 1 and 2 consent documentation were in use (April 1998–October 2008) was granted in January 2025, effective from January 2024. As shown in Figure 2b, 878 and 868 participants were recruited to the Cancer Biobank using versions 1 and 2 consent documentation, respectively. These figures exclude any participants who had been reconsented using later (HRR/GDPR compliant) Cancer Biobank consent documentation.

## Discussion

Work to accurately and transparently present numbers of participants, samples and proof of consent in the Cancer Biobank is ongoing. This slow, detailed work involves cross-checking paper files for evidence of consent forms with electronic patient records in the Cancer Biobank LIMS system. Prior to 2019, consent forms were signed in single copy only, with provision of a LIMS tick-box to indicate that participant consent had been obtained. This was because earlier consent forms were occasionally sent to the patient’s chart rather than back to the research laboratory for storage. Any consent forms that were identified during this process were scanned for upload to the LIMS system. All files were additionally checked for evidence of the most recent consent form. As most patients attend hospital for follow-up, many had been reconsented using later version(s) of consent documentation.

The issue of reconsenting participants for whom no proof of consent could be demonstrated was raised during the consent declaration process. We concluded that while reconsenting patients might be ideal, there were compelling reasons that could make the process impractical. Ethics approval to re-contact participants (for the purpose of reconsent) was not routinely included on early versions of consent documentation. As a solution, a plan to approach participants who continued to attend follow-up clinics was proposed. For this reason, the Cancer Biobank continues to manually check each of the approximately 9000 participant records for updated consent documentation. We estimate that approximately 70 participants during the 1998–2005 timeframe were subsequently reconsented using later consent documentation.

A workflow was developed to approach the issue of reconsenting participants that had been recruited between 1998–2005 using versions 1 and 2 consent documentation. We conducted an exercise, randomly selecting 7 former breast cancer patients who donated tissue samples during the timeframe. Only 1 of these patients was still attending Galway University Hospital, as of July 2024.

Distress for patients, or their relatives was also highlighted as a reason to avoid reconsent. Mee *et al.* (2021) ^8^ reported on a study to retrospectively obtain consent from nearly 2,000 breast cancer patients diagnosed between 2004 and 2014 for the multi-institutional Breast Predict study. Former patients were contacted via their general practitioners, who on 104 occasions, advised against contacting some patients due to health or emotional reasons. Of those contacted, 76% responded, but 22% of responses were excluded due to incomplete or inaccurate forms. Ultimately, only 399 patient samples (about 20% of the original cohort) were included, highlighting challenges of recontacting and retrospective consenting in similar-sized cohorts.

## Conclusions

Prior to the introduction of triplicate copies of consent forms and digitising signed forms to the Cancer Biobank LIMS in 2019, the proportion of participants with no verifiable consent documentation on file averaged 24%, highlighting gaps in record-keeping up to that point. A consent declaration has enabled the Cancer Biobank to continue to store personal data, while efforts to obtain participant reconsent during clinic visits continue. Efforts to recover missing consent documentation have been partly successful however manual chart reviews have only yielded small improvements in overall figures. Another challenge with archival consent documentation was the propensity of errors (e.g. incomplete signing, erroneous entries), underlining the need for improved consent data management.

The Cancer Biobank’s detailed review and continuous auditing emphasise the complexity of maintaining valid consent documentation longitudinally for long-term studies using biobanked samples. While legacy consent issues raise ethical and legal challenges, the scientific benefits of responsibly using these samples can outweigh the risks if proper safeguards and approvals are in place. Efforts to resolve consent issues including reconsenting participants and seeking waivers when recontact is impractical, can enable continued use of these samples while respecting participant rights. Ignoring legacy samples due to consent concerns could impede the use of important longitudinal data that enable understanding of disease progression and long-term outcomes, which are essential for developing improved diagnostics and personalised medicine.

## Ethics and consent

This study was conducted as part of Cancer Biobank quality management programme under Clinical Research Ethics Committee approval Ref. C.A. 2762 (Cancer Biobank Quality Management and accreditation). For Legacy participants recruited under earlier versions (1998–2008), ongoing storage and use of personal data are covered under a Consent Declaration granted by the Health Research Consent Declaration Committee (HRCDC) in 2024.

## Data Availability

The data underlying this study consist of historical participant consent records held by the Cancer Biobank at the University of Galway and HSE West and North West. Due to the inclusion of personally identifiable and sensitive health information, these records will not be publicly shared. Aggregated data supporting the findings of this article are available within the manuscript. Researchers who wish to request access to aggregated or pseudonymised data for justified research purposes may contact the Cancer Biobank at adminbiobank@universityofgalway.ie. Access will be considered on a case-by-case basis and may be subject to institutional data sharing agreements and/or ethical approval.
