# Supplementary material for: Documenting participant Consent in archival records: Aligning yesterday's practices with today's processes
Source: HRB Open Res. 2025 Oct 15;8:110. [Version 1] doi: 10.12688/hrbopenres.14254.1 (PMC12712454; doi:10.12688/hrbopenres.14254.1)
Supplement: Peer review history

# Reviewer report: Reviewer response for version 1

**DOI:** 10.21956/hrbopenres.15676.r52075

**Published:** 2025-12-17

**Authors:** Wayne Ng

**Affiliations:** 1 Victorian Cancer Biobank, Melbourne, Australia

This is an important article that report on consent verification to address ethics and legal compliance issues. It is transparent in the process and provide a reference for the scientific community on approaches to address such issues. 

 The article also emphasised the cost and resources spent to go through the whole process which should significantly be considered for similar incidents by other biobanking initiatives. This should also be planned and prepared for any new collections moving forwards. 

 While the article mentioned about the consent declaration for storing the data of participants, it is not clear to whether the samples and data without proof consent document are still allowed to be used for research? If not, what's the purpose of storing these data.

Is the work clearly and accurately presented and does it cite the current literature?

Yes

If applicable, is the statistical analysis and its interpretation appropriate?

Yes

Are all the source data underlying the results available to ensure full reproducibility?

No

Is the study design appropriate and is the work technically sound?

Yes

Are the conclusions drawn adequately supported by the results?

Yes

Are sufficient details of methods and analysis provided to allow replication by others?

Yes

Reviewer Expertise:

cancer, biobanking

I confirm that I have read this submission and believe that I have an appropriate level of expertise to confirm that it is of an acceptable scientific standard.

# Reviewer report: Reviewer response for version 1

**DOI:** 10.21956/hrbopenres.15676.r50811

**Published:** 2025-11-04

**Authors:** Jacqueline James

**Affiliations:** 1 Regional Molecular Diagnostic Service, Belfast Health and Social Care Trust, Belfast, UK

This is a well written and timely article focused on the Cancer Biobank at the University of Galway.  The study aimed to robustly audit and verify consent documentation over an extended period of time (1998-2025). The study design utilised a mixed method approach combining manual chart reviews and electronic record checks.  Participant data was digitised and pseudonymised using the Biobank LIMS system, with missing consents investigated through chart reviews and hospital archives.  Out of 8,925 participants, 65% had valid Cancer Biobank consent, 13% had other study consents, and 21% had no documented proof. Audits and cross-checks ensured compliance, and a consent declaration was granted for early participants lacking documentation.  Before 2019, 24% of participants lacked verifiable consent due to gaps in record keeping. Reconsenting older participants proved difficult due to ethical and emotional concerns. These results add to the existing literature and highlight the challenges of retrospective consent in long-term studies. Access to the raw data used in the analysis would require appropriate institutional approvals.

 In summary, since its establishment in 1998 the Cancer Biobank at the University of Galway has underpinned international breast cancer research, contributed to improved genetic screening and has aligned with GDPR and biobanking standards. In 2025 a comprehensive review of records from over twenty years of Biobank operations examined consent documentation for regulatory and ethical compliance. The results have real-world relevance and address practical challenges in retrospective consent and data governance for Biobanks in Ireland.  However, Consent declarations and safeguards put in place at the Cancer Biobank at the University of Galway now support the ethical use of valuable samples, enabling progress in personalised medicine and long-term research. 

 There are no required changes to the article.

Is the work clearly and accurately presented and does it cite the current literature?

Yes

If applicable, is the statistical analysis and its interpretation appropriate?

Not applicable

Are all the source data underlying the results available to ensure full reproducibility?

Yes

Is the study design appropriate and is the work technically sound?

Yes

Are the conclusions drawn adequately supported by the results?

Yes

Are sufficient details of methods and analysis provided to allow replication by others?

Partly

Reviewer Expertise:

Biobanking, Cellular and Digital Pathology, Molecular Diagnostics

I confirm that I have read this submission and believe that I have an appropriate level of expertise to confirm that it is of an acceptable scientific standard.
